# Enhanced Locomotor Activity Is Required to Exert Dietary Restriction-Dependent Increase of Stress Resistance in *Drosophila*

**DOI:** 10.1155/2015/813801

**Published:** 2015-04-28

**Authors:** Saurav Ghimire, Man Su Kim

**Affiliations:** College of Pharmacy, Inje University, Gimhae 621-749, Republic of Korea

## Abstract

Dietary restriction (DR) is known to be one of the most effective interventions to increase stress resistance, yet the mechanisms remain elusive. One of the most obvious DR-induced changes in phenotype is an increase in locomotor activity. Although it is conceptually perceivable that nutritional scarcity should prompt enhanced foraging behavior to garner additional dietary resources, the significance of enhanced movement activity has not been associated with the DR-dependent increase of stress resistance. In this study, we confirmed that flies raised on DR exhibited enhanced locomotive activity and increased stress resistance. Excision of fly wings minimized the DR-induced increase in locomotive activity, which resulted in attenuation of the DR-dependent increase of stress resistance. The possibility that wing clipping counteracts the DR by coercing flies to have more intake was ruled out since it did not induce any weight gain. Rather it was found that elimination of reactive oxygen species (ROS) that is enhanced by DR-induced upregulation of expression of antioxidant genes was significantly reduced by wing clipping. Collectively, our data suggests that DR increased stress resistance by increasing the locomotor activity, which upregulated expression of protective genes including, but not limited to, ROS scavenger system.

## 1. Introduction

Aging is a multifaceted process that alters organism's structural architecture and functional capacity over the course of life. Unfortunately, aging-associated phenotypical and cellular changes often lead to a decrease in capability for daily tasks while susceptibility to many diseases such as cardiovascular disorders, cancer, and dementia is increased [1–3]. Over the last few decades, an aspiration to revert the detrimental consequences of aging has fueled numerous research projects to find out that dietary restriction (DR) is the most reliable and conserved manipulation to render organisms more competent against environmental stress. DR is the key to prolong the lifespan and delay the clinical onset and progression of the aforementioned formidable diseases [4–6].

DR reduces nutrient intake without inducing malnutrition [7]. However, cellular mechanisms by which DR conveys such beneficial outcomes are not yet completely explored. In addition to metabolic conversion towards more efficient energy utilization from limited dietary sources [8–10], DR is shown to increase the locomotor activity in a variety of species spanning from flies to primates [11–13]. Although it is conceptually perceivable that nutritional scarcity should prompt enhanced foraging behavior to garner additional dietary resources, the significance of increased activity is not yet linked to the DR-dependent increase of stress resistance.

We used* Drosophila* as an* in vivo* animal model to explore the importance of DR-induced increased locomotor activity in the organism's ability to cope with environmental stress. Apart from the ease of culturing in laboratory settings and the proven track record of providing insight into human diseases [14],* Drosophila*'s relatively short lifespan let us monitor the significance of locomotor activity over the entire course of life. Using the wing excision technique, which successfully attenuated the DR-induced increase in locomotor activity [15], we demonstrated that enhanced locomotor activity is required to exert the DR-dependent increase of stress resistance, at least in part, via the induction of the reactive oxygen species scavenger system. Living not just longer but healthier is an ultimate research goal for gerontology and the response of the organism to stress would be an indicator to estimate its quality of health. Therefore, we envision that this study would usher new lines of stress resistance studies, eventually paving the avenue to develop novel interventions that reduce the risk of cancer, dementia, and diabetes in the elderly.

## 2. Materials and Methods

### 2.1. Fly Husbandry

Adult* w1118* strain obtained from Bloomington* Drosophila* Stock Center (Bloomington, IN, USA) was used for the experiments performed in this study. As described previously [16], the flies were kept in a plastic bottle containing a standard* Drosophila* diet (8.5 g corn meal, 5 g sugar, 1.5 g live yeast, and 0.46 g agar in 100 mL distilled water; final 1% v/v concentration of propionic acid was added later to prevent mold growth) in a light (12-hour light/dark cycle), temperature (25°C), and humidity (60%) controlled fly incubator. Flies were transferred to fresh bottles approximately every 3 weeks to refresh the stock. Unless otherwise specified, all experiments were performed in the fly incubator.

### 2.2. Crossings, Diet Preparation, and Wing Clipping

10 virgin female flies and 3–5 male flies were kept together inside bottles containing a standard* Drosophila* food for 4 days, which allows female flies to have a sufficient time to mate and lay eggs. After that, parents were discarded and eggs were hatched to larvae, which metamorphose into the adult fly through the pupae stage. Although most flies eclosed 10 days after crossings in our experimental condition, we sorted out female flies 14 days after crossings in order to collect only age-matched mated female flies for this study. Female flies were used throughout experiments since phenotype changes induced by dietary restriction have been shown to be much greater in female flies than in male files [17]. CO_2_ was used to temporary anesthetize flies for sorting and wing clipping. Sorted female flies were fed on either rich diet (*ad libitum*, AL) or restricted diet (dietary restriction, DR). The recipes for the preparation of AL and DR are the same as the standard* Drosophila* diet with the exception that 1.5% live yeast was replaced with 5% or 0.5% yeast extract for AL or DR, respectively. For wing clipping, both sides of fly wings were manually removed with fine tweezers. Sorting and wing clipping were completed within 3 min to minimize CO_2_ toxicity.

### 2.3. Measurement of Locomotor Activity

Flies at the age of 10 days were transferred to new polystyrene vials (25 mm diameter) that contain freshly made DR or AL diets. Then, the vials were placed into the* Drosophila* LAM25 Locomotor Activity Monitor (Trikinetics, Waltham, MA, USA) and data were acquired and processed with DAMSystem 308 software (Trikinetics, Waltham, MA, USA).

### 2.4. Stress Resistance Assays

To examine flies' ability to resist environmental stresses, starvation and reactive oxygen species (ROS) stress assays were performed. For the starvation stress assay, flies at the age of 10 days were transferred to starvation media made of 1% agar. Survivorship curves were constructed after scoring dead flies every 4 hours. Flies were transferred to fresh starvation media every day and dead flies were eliminated when possible. For ROS stress assay, 10-day-old flies were transferred to either AL or DR diet supplemented with 0.2 mM paraquat. Survivorship curves were generated by scoring dead flies every day. The flies were transferred to fresh paraquat supplemented diets every other day.

### 2.5. RNA Preparations, cDNA Synthesis, and Quantitative Polymerase Chain Reaction (qPCR)

To compare gene transcription levels, qPCR was performed. According to the manufacturer's protocols total RNAs were isolated from five flies using RNeasy Mini Kit (Qiagen, Valencia, CA, USA) and cDNA was prepared from the isolated total RNA with QuantiTect Reverse Transcription Kit (Qiagen, Valencia, CA, USA). To quantify the amount of transcripts, SYBR Green based qPCR was performed with SensiFast SYBR NO-Rox Kit (Taunton, MA, USA) using a Rotor-Gene Q thermocycler (Qiagen, Valencia, CA, USA) under the following conditions: 95°C, 5 seconds for denaturation, 55°C, and 20 seconds for annealing and extension. The following gene specific primer sets were used: *β-tubulin* (CG9277) F, ACA-TCC-CGC-CCC-GTG-GTC, R, AGA-AAG-CCT-TGC-GCC-TGA-ACA-TAG;* superoxide dismutase 1* (*SOD1*, CG11793), F, CAA-GGG-CAC-GGT-TTT-CTT-C, R, CCT-CAC-CGG-AGA-CCT-TCA-C;* catalase* (CG6871), F, TGA-CTA-CAA-AAA-CTC-CCA-AAC-G, R, TTG-ATT-CCA-ATG-GGT-GCT-C; and* phospholipid hydroperoxide glutathione peroxidase* (*PHGPx*, CG12013), F, GAC-ATC-GGC-GAG-GTG-TTC, R, ACT-TGG-TGA-AGT-TCC-ACT-TGA-TT. The specificity of amplicons was verified with a melting curve analysis and the messenger levels were normalized using *β*-tubulin as an internal control and calculated according to the ΔΔCt method [18].

### 2.6. Reagents

*Drosophila* diets (corn meal, sugar, live yeast, yeast extract, and agar) were purchased from Hansol Tech Inc. (Seoul, Korea). Propionic acid (catalog # 64655-0430) and paraquat (catalog # 856177) were obtained from Junsei Chemical Co. Ltd. (Tokyo, Japan) and Sigma-Aldrich (St. Luis, MO, USA), respectively.

### 2.7. Statistics

Log-rank test was used for testing significance between survivorship curves. Unpaired two-tailed Student' *t*-test and ANOVA with* Bonferroni* post hoc test were used for the statistical comparison between two independent groups and more than two independent groups, respectively.

## 3. Results

### 3.1. Wing Clipping Attenuates the DR-Induced Increase in Locomotor Activity

**Figure 1:**
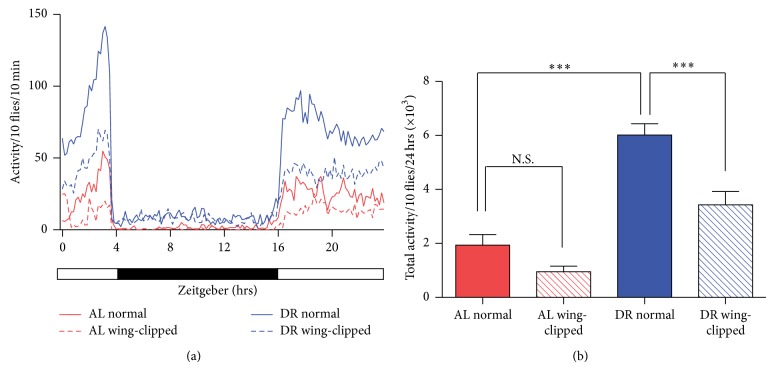
Wing clipping attenuates an increase of locomotor activity induced by dietary restriction in* Drosophila*. (a) In each experimental group, normal or wing-clipped flies were reared on either* ad libitum* (AL, 5% YE) or restricted (DR, 0.5% YE) diet. At the age of 10 days, flies' spontaneous locomotor activity was measured from each group with 10 min binning, which was plotted as a function of time. Each solid and dotted line represents mean locomotor activity calculated from 8 independent experiments. The white and black bars indicate the light and dark cycle where flies are housed. (b) The data presented in (a) was replotted as a bar graph to demonstrate flies' total spontaneous locomotor activity for 24 hrs. Data were presented as mean ± S.E.M. ^∗∗∗^ *P* < 0.001; N.S. stands for nonsignificant. One-way ANOVA with* Bonferroni* post hoc test.

Previous reports demonstrated that flies reared on DR showed higher locomotor activity than those grown on AL [15]. We were able to reproduce this DR-induced phenotype change in our experimental settings. Newly eclosed age-matched flies were grown on either AL or DR condition. At the age of 10 days, flies reared on these two different diet conditions were transferred to* Drosophila* Locomotor Activity Monitor and their spontaneous activities were measured in 10-minute bins for 24 hours. As reported, DR drastically increased flies' total locomotor activity (Figure 1(b)). Notably, flies demonstrated rhythmic locomotor activity (Figure 1(a)). Although their locomotor activity was much greater in light (ZT0–ZT4 and ZT16–ZT24, which corresponded to 4pm–8pm and 8am–4pm, resp.) than off light (ZT4–ZT16, which corresponded to 8pm–8am), characteristic peak activities were observed twice per a 24-hour period, at approximately ZT2–4 and ZT18–20.

To examine physiological effects of higher locomotor activity in flies reared on DR, we attenuated the DR-dependent increase in locomotor activity. To achieve this, we clipped off both wings with fine tweezers while flies were being sorted. Despite that, flies were viable and did not show any abnormal behavior. To verify that wing clipping indeed reduces locomotor activity, normal and wing-clipped flies were reared on DR for 10 days. Then, their spontaneous locomotor activities were monitored for 24 hours, which revealed significant reduction of locomotor activity in wing-clipped groups. The extent of reduction in locomotor activity by wing clipping was more obvious for flies fed on DR than those reared on AL probably due to the low baseline locomotor activity of normal flies reared on AL. This implies that wing clipping may not be able to further decrease the movement of flies raised on AL. Collectively, it was confirmed that DR increases locomotor activity and wing clipping can be used as a tool to diminish the DR-induced increase in locomotor activity.

### 3.2. Wing Clipping Does Not Induce Weight Gain

**Figure 2:**
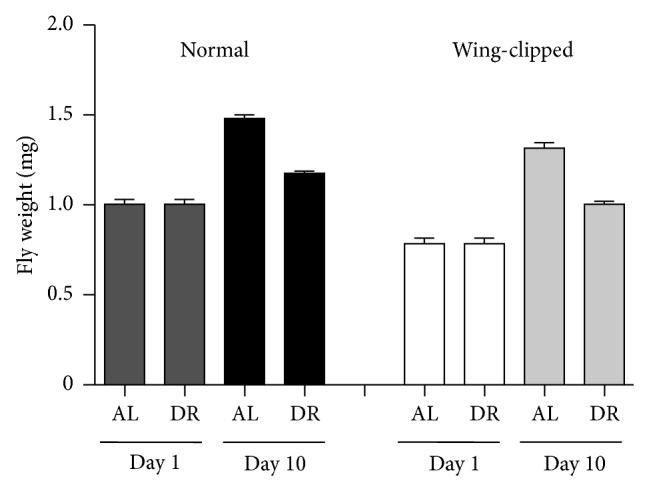
Wing clipping does not induce weight gain. Eclosed normal adult flies were reared either on restricted diet (DR, 0.5% YE) or* ad libitum* (AL, 5% YE). At the age of 1 day and 10 days, fly weight in each group was measured. The effect of wing clipping on weight gain was examined at the age of 1 day and 10 days by measuring whole body weight of wing-clipped flies reared in the same condition as for the normal flies. Data are represented as mean ± S.E.M, *n* = 5 for each condition.

Reduction in flies' locomotor activity by wing clipping coerced flies to further stay around food, which may increase the food consumption. We performed the wing clipping to specifically abolish the DR-induced increase in locomotor activity without interrupting other DR-associated changes such as metabolisms. Therefore, it was necessary to vindicate that wing clipping did not affect patterns of food intake. As a simple method, we measured flies' weight at days 1 and 10 to examine if wing-clipped flies had received more food. As expected, normal flies had a comparable weight gain at days 1 and 10. The rate of weight gain was much faster in flies reared on AL than those on DR for being on the rich diet. However, wing clipping did not affect flies' weight (Figure 2). Wing-clipped flies were slightly lighter than normal flies at day 1 due to the excision of wings. This trend continued when the weight was measured at day 10, suggesting that wing clipping had not driven flies to take more food. Therefore, we decided to proceed to investigate the effects of increased locomotor activity on flies' physiology using the wing clipping method.

### 3.3. Wing Clipping Decreases Flies' Ability to Resist Starvation Stress

**Figure 3:**
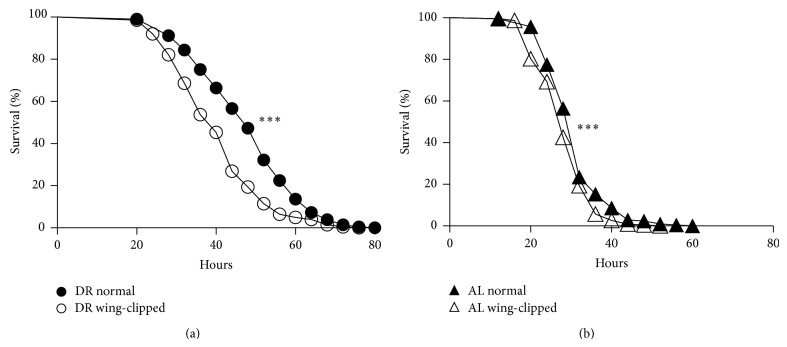
Wing clipping decreases flies' ability to resist starvation stress. (a) Normal (closed circles) and wing-clipped (open circles) adult flies were reared on restricted diet (DR, 0.5% YE). At the age of 10 days, flies were transferred to 1% agar media to examine their ability to resist starvation stress. Survivorship curves were built by scoring dead flies every 4 hours. (b) The same experiment as described in (a) was performed with the exception that normal (closed triangles) and wing-clipped (open triangles) adult flies were reared on* ad libitum* (AL, 5% YE) condition. Log-rank test was used to determine statistical significance. ^∗∗∗^ *P* < 0.001, *n* = 201–228 for each group.

DR is shown to increase organisms' ability to resist environmental stress. To probe the significance of DR-induced increased locomotor activity on enhancing the ability to withstand stress, we monitored flies' responsiveness to starvation with wing clipping. Normal and wing-clipped flies maintained on either DR or AL were subjected to the starvation assay at the age of 10 days. As shown in Figure 3(a), median survival time of normal flies reared on DR was 48 hours while that of wing-clipped ones raised on the same diet condition was only 40 hours (17% reduction). In flies raised on AL, a statistically significant yet minimal decrease was observed in median survival time under starvation condition in wing-clipped flies (Figure 3(b)). It is in good agreement with the previous observation that showed an insignificant effect for wing clipping on reducing spontaneous activity for flies raised on AL (Figure 1). Collectively, these observations suggest that the increase in locomotor activity contributed to DR-induced resistance against starvation stress.

### 3.4. Wing Clipping Decreases Flies' Ability to Resist ROS Stress by Reducing ROS Scavenger Gene Expression

**Figure 4:**
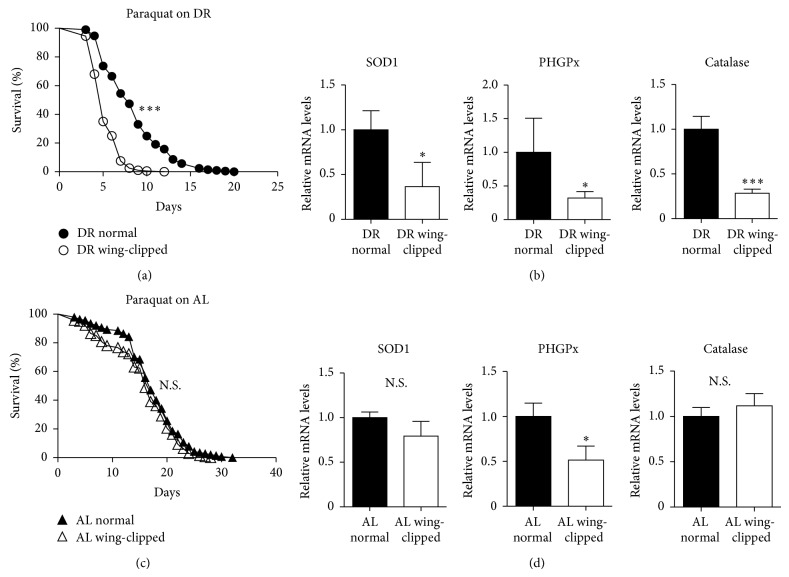
Wing clipping makes flies fed on restricted diet (DR) vulnerable to ROS stress. (a) After rearing normal (closed circles) and wing-clipped (open circles) adult flies on restricted diet (DR, 0.5% YE) for 10 days, flies in both groups were transferred to DR diets supplemented with 0.2 mM paraquat. Survivorship curves were generated by scoring dead flies every day when transferring live flies to freshly made 0.2 mM paraquat supplemented DR media. (b) Relative mRNA levels of* SOD1*,* PHGPx*, and* catalase* were calculated using 2^−ΔΔCt^ method. SYBR Green based qPCR was performed with cDNA samples prepared from normal (black bars) or wing-clipped (open bars) flies grown on DR diet for 10 days. (c-d) The same experiments as described in (a-b) were performed with the exception that normal (closed triangles, black bars) and wing-clipped (open triangles, open bars) adult flies were fed* ad libitum* (AL, 5% YE) for 10 days, which was subject to either ROS stress assay by transferring flies to AL diets supplemented with 0.2 mM paraquat as shown in (a) or gene expression assay as described in (b). Log-rank test for (a) and (c). Two-tailed Student's *t*-test for (b) and (d). ^∗∗∗^ *P* < 0.001, ^∗^ *P* < 0.05. N.S. stands for not significant. *n* = 140–209 for (a) and (c). qPCR performed twice using biologically independent RNA preparations generated consistent results. Data was represented as mean ± ranges.

Next, we performed an ROS stress assay as an additional approach to evaluate flies' responsiveness to stress. Normal and wing-clipped flies were reared on either DR or AL diets. At the age of 10 days, flies were challenged with ROS by supplementing diets with paraquat and survivorship curves were generated by scoring dead flies every day. As shown in Figure 4(a), wing clipping shortened survival in flies raised on DR while it did not influence flies reared on AL (Figure 4(c)). To explain these observations at the molecular level, quantitative PCR was performed at the age of 10 days to assess if expression of genes that were involved in ROS scavenger system was altered by wing clipping. In flies reared on DR, wing clipping significantly diminished messenger levels of all major ROS scavenger genes. These genes are* superoxide dismutase 1* (*SOD1*),* phospholipid hydroperoxide glutathione peroxidase* (*PHGPx*), and* catalase* (Figure 4(b)). On the contrary, wing clipping only reduced* PHGPx* transcript for flies reared on AL (Figure 4(d)). Taken together, these findings imply that wing clipping made DR-raised flies susceptible to ROS stress by decreasing scavenger gene expression probably due to a decrease in locomotor activity.

## 4. Discussion

Aging is often regarded as an irreversible biological process characterized by a decrease in withstanding stresses, increasing the susceptibility to numerous diseases. Yet, rapid progress in gerontology has revealed that aging is a malleable process that can be significantly slowed through different manipulations [19]. An important example is dietary restriction that is widely believed to be the most promising approach that robustly increases the resistance to toxic substances and numerous forms of stress with incompletely understood mechanisms [20]. In this study, we provided evidence supporting the hypothesis that DR-induced enhanced locomotor activity renders organisms more capable of resisting environmental stress (Figures 3 and 4). At the molecular level, this could be achieved, at least in part, through the induction of ROS scavenger system (Figure 4). DR has shown to trigger numerous phenotypical and molecular changes [21]. We believe the novelty of our research lies in associating two important DR-induced alternations, namely, enhanced movement activity and induction of ROS scavenger system. This can partially explain the health promoting effect of DR.

From the technical perspective, wing clipping was utilized to attenuate the DR-induced increase in locomotor activity (Figure 1). Mechanical detachment of fly wings with tweezers could have imposed damage on flies. Therefore, we carefully monitored flies' behavior to ensure that our experimental approach is appropriately employed. Despite a reduction in locomotor activity by this technique, flies' rhythmical movement activity was not perturbed (Figure 1(a)). Furthermore, no significant change in feeding behavior was observed, resulting in an expected weight gain for the wing-clipped flies (Figure 2). Thus, although only occasionally used by others thus far [15], we found wing clipping simple yet effective to make it worthwhile in studies using movement assays. As a complimentary approach, we believe it would be interesting to examine changes in stress responsiveness with a tool that could enhance locomotor activity in flies reared on AL in the future. Yet, developing a solid method that boosts spontaneous activity without disturbing the normal biology is more challenging.

Although our focus was on ROS scavenger system, it does not rule out other mechanisms that can be involved in DR-induced enhanced stress resistance. The employed starvation assay prevents flies from access to nutritional source without causing dehydration (Figure 3). Under this harsh condition, it has been suggested that survival was determined by the ability to efficiently use reserved energy sources. In particular, DR-induced altered fat metabolism is ascribed as a critical factor that modifies flies' capability to resist starvation stress [22, 23]. Elevated steady state triglyceride levels are observed under DR conditions in flies [24]. Interestingly, long-lived TOR (target of rapamycin) and ILS (insulin-like signaling) mutants also show increased triglyceride levels [25, 26]. This is because at least a partial response in fat mobilization under DR conditions is mediated through TOR and ILS pathways. The mechanism of reorganization in fat metabolism appears to be conserved across species enabling them to cope with harsh conditions such as DR. This is shown in a study on rodents that found a correlation between maintaining adipose levels and lifespan extension under DR [27]. Thus far, several groups have used the genomic approach to reveal the comprehensive picture of alteration in the gene expression profile under DR conditions [15, 28, 29]. Although the picture is not yet complete, accumulated evidence suggests an increase in expression of genes involved in fatty-acid synthesis [28, 29]. Therefore, it is plausible that DR-induced enhanced locomotor activity could affect the expression patterns of genes that are associated with fat metabolism.

Another intriguing question is, what is the driving force that keeps the high level of locomotor activity despite the DR-induced reduction in total calorie intake? In this regard, Katewa et al. [15] recently propose that fatty-acid synthesis and breakdown in the muscle are facilitated by DR, which emphasizes that high levels of lipid turnover, not just total fat contents, are critical for generating sufficient ATP that is required for sustaining elevated muscular activity. Also, we have found that DR causes a change in the composition of ion (i.e., Na^+^ and K^+^) in fly's hemolymph (unpublished data), which may alter the excitability of the contractile unit composed of muscular cells and neurons. In agreement with our preliminary observations, low calorie diet has been reported to increase neurotransmission at neuromuscular junction, which may contribute to enhanced motor function [30]. Further research on examining the driving force that triggers enhanced locomotor activity would help elucidate the DR-induced cellular changes.

From clinical viewpoint, the importance of maintaining a higher level of locomotor activity for improved stress resistance should be mentioned. Accumulated body of literature unambiguously concludes that sarcopenia is closely related to increased morbidity and mortality both in healthy individuals and in patients [31]. This implies that muscle is not a simple organ with contractility but a complex structure that actively interacts with other organs. Recent research proposes that muscle generates myokines, muscle-derived anti-inflammatory substances, which affect the functions of structurally unconnected body parts such as adipocytes, endothelial cells, and the immune system [32]. Therefore, induction of ROS scavenger system by enhanced locomotor activity could help preserve muscular integrity, which leads to generation of myokines that protect the organism from environmental stress.

Finally, oxidative stress has been regarded as the main culprit that accelerates aging. Apart from directly causing damage to macromolecules such as nucleic acids, proteins, and lipids, ROS can initiate cellular signaling pathways that contribute to disease. For instance, growth factors trigger cellular proliferative cascades via mitogen-activated protein kinase (MAPK) and phosphoinositol 3-kinase (PI3K) pathways, both of which are further activated by ROS, resulting in sustained growth signals. This effect is postulated to underlie aberrant proliferation in tumorigenesis [33]. However, organisms are armed with a cellular machinery to counteract oxidative stress. A well-recognized cytoprotective mechanism is mediated by vitagenes, which include thioredoxin system and heat shock proteins (hsp 32 aka heme oxygenase-1 and hsp 70) [34, 35]. Collectively, they restore ROS balance and serve as molecular chaperones repairing or eliminating damaged proteins. Therefore, it is worthwhile to examine if enhanced locomotor activity is required to upregulate the expression of vitagenes in maintaining the redox homeostasis and minimizing the accumulation of misfolded proteins.

In conclusion, we anticipate that our study would provide mechanistic insight into the DR effects that would help develop practical interventions to improve the senile quality of life by mimicking the DR-induced cellular changes.

## 5. Conclusions

Dietary restriction (DR) strengthens the organism against environmental stresses through poorly characterized mechanisms. In our study, DR-enhanced locomotor activity was experimentally reduced by wing clipping. This manipulation did not affect the pattern of food intake, which was indirectly measured by weight gain. However, attenuation of the DR-enhanced locomotor activity decreased the flies' resistance against environmental stresses. Furthermore, we found that the level of antioxidant gene transcripts was significantly reduced in wing-clipped flies reared on DR. Therefore, we suggest that upregulation of the ROS scavenger system by a DR-induced increase in locomotor activity may serve as a molecular basis to exert the beneficial effect of DR.
